# Supplementary material for: Structural Achievability of an NH–π Interaction between Gln and Phe in a Crystal Structure of a Collagen-like Peptide
Source: Biomolecules. 2022 Oct 6;12(10):1433. doi: 10.3390/biom12101433 (PMC9599227; doi:10.3390/biom12101433)
Supplement: Supplementary file 1 [file biomolecules-12-01433-s001.zip › biomolecules-1915619-supplementary.pdf]

Methods

Calculation of geometric parameters

In order to calculate six geometric parameters, d_{NM}, θ, ω, $d_{\mathrm{C\gamma\text{-}\pi}}$, τ, χ, the coordinate sets including (1) the N and H atoms in one of the two amide NH groups of Gln or acetamide with shorter distance to the phenyl ring, (N_x, N_y, N_z) and (H_x, H_y, H_z), (2) the Cγ atom of Gln, $(C_{\gamma,x}, C_{\gamma,y}, C_{\gamma,z})$, (3) all atom of the phenyl ring of Phe, $(C_{i,x}, C_{i,y}, C_{iz})$, where $i = \gamma, \delta_1, \delta_2, \varepsilon_1, \varepsilon_2, \zeta$ were retrieved from the pdb files. (3) the Cγ' $(C'_{\gamma,x}, C_{\gamma,y}', C_{\gamma,z}')$ and N' (N_x', N_y', N_z') were the point projected to the plan. Since hydrogen atoms are missing from most pdb files, theoretical positions of hydrogen atoms (X-H=1.0 Å) were then added with HGEN software, but only those at positions allowing this calculation unambiguously were used further. The mass center and normal vector of the phenyl ring, (M_x, M_y, M_z) and $\vec{n}$, and five vectors, $\overrightarrow{MN}$, $\overrightarrow{HM}$, $\overrightarrow{HN}$, $\overrightarrow{C\gamma N}$, $\overrightarrow{MC\gamma}$ were defined as

$$(M_x, M_y, M_z) = \frac{1}{6}\sum (C_{ix}, C_{iy}, C_{iz}) \qquad (1)$$

$$i = \gamma, \delta_1, \delta_2, \varepsilon_1, \varepsilon_2, \zeta$$

$$\overrightarrow{C\gamma C\varepsilon_j} = \left(C_{\varepsilon_j,x}, C_{\varepsilon_j,y}, C_{\varepsilon_j,z}\right) - (C_{\gamma,x}, C_{\gamma,y}, C_{\gamma,z}) \qquad (2)$$

$$j = 1, 2$$

, where $\overrightarrow{C\gamma C\varepsilon_j}$ is the vector point from $C\gamma$ atom to $C\varepsilon_1$ (j=1) or $C\varepsilon_2$ (j=2) of the Phe.

$$\vec{n} = \overrightarrow{C\gamma C\varepsilon_1} \times \overrightarrow{C\gamma C\varepsilon_2} \qquad (3)$$

$\vec{n}$ is the normal vector of the phenyl ring of Phe.

$$\overrightarrow{MN} = (N_x, N_y, N_z) - (M_x, M_y, M_z) \qquad (4)$$

$$\overrightarrow{HN} = (N_x, N_y, N_z) - (H_x, H_y, H_z) \qquad (5)$$

$$\overrightarrow{HM} = (M_x, M_y, M_z) - (H_x, H_y, H_z) \qquad (6)$$

$$\overrightarrow{C\gamma N} = (N_x, N_y, N_z) - (C_{\alpha,x}, C_{\alpha,x}, C_{\alpha,x}) \qquad (7)$$

$$\overrightarrow{MC\gamma} = (C_{\gamma,x}, C_{\gamma,x}, C_{\gamma,x}) - (M_x, M_y, M_z) \qquad (8)$$

$$\overrightarrow{C\gamma' N'} = (N_x', N_y', N_z') - (C_{\gamma,y}', C_{\gamma,y}', C_{\gamma,z}') \qquad (9)$$

$$\overrightarrow{C\beta C\gamma} = (C_{\gamma,x}, C_{\gamma,x}, C_{\gamma,x}) - (C_{\beta,x}, C_{\beta,x}, C_{\beta,x}) \qquad (10)$$

Three distance parameters, d_{NM}, d_{HM}, $d_{\mathrm{C\alpha\text{-}\pi}}$, were defined as:

$$d_{NM} = |\overrightarrow{NM}| \qquad (11)$$

$$d_{C\alpha-\pi} = \frac{|\overrightarrow{MC\alpha} \cdot \vec{n}|}{|\vec{n}|} \qquad (12)$$

Three angle parameters, θ, ω, τ, χ were defined as:

$$\theta = \left|\arccos\left(\frac{\overrightarrow{NM} \cdot \vec{n}}{|\overrightarrow{NM}||\vec{n}|}\right)\right| \quad (0° < \theta < 90°) \qquad (13)$$

$$\omega = \arccos\left(\frac{\overrightarrow{HN} \cdot \overrightarrow{HM}}{\left|\overrightarrow{HN}\right|\left|\overrightarrow{HM}\right|}\right) \quad (0° < \omega < 180°) \tag{14}$$

$$\tau = \arcsin\left(\frac{\overrightarrow{C\gamma N} \cdot \vec{n}}{\left|\overrightarrow{C\gamma N}\right|\left|\vec{n}\right|}\right) \quad (-90° < \tau < 90°) \tag{15}$$

$$\chi = \arccos\left(\frac{\overrightarrow{C\gamma' N'} \cdot \overrightarrow{C\beta C\gamma}}{\left|\overrightarrow{C\gamma' N'}\right|\left|\overrightarrow{C\beta C\gamma}\right|}\right) \quad (0° < \chi < 180°) \tag{16}$$

θ is the acute angle (0° < θ ≤ 90°) between the vector $\overrightarrow{NM}$ and the normal vector $\vec{n}$. ω is the angle between the vector $\overrightarrow{NH}$ and $\overrightarrow{HM}$. τ is the angle between the $\overrightarrow{C\gamma N}$ and the phenyl plane. If Cγ is located in the same direction as the normal vector, $\vec{n}$, of the phenyl ring, it is positive (0° < τ < 90°), otherwise, it is negative (-90° < τ < 0°). χ is the angle between the vector $\overrightarrow{C\gamma' N'}$ and $\overrightarrow{C\beta C\gamma}$.

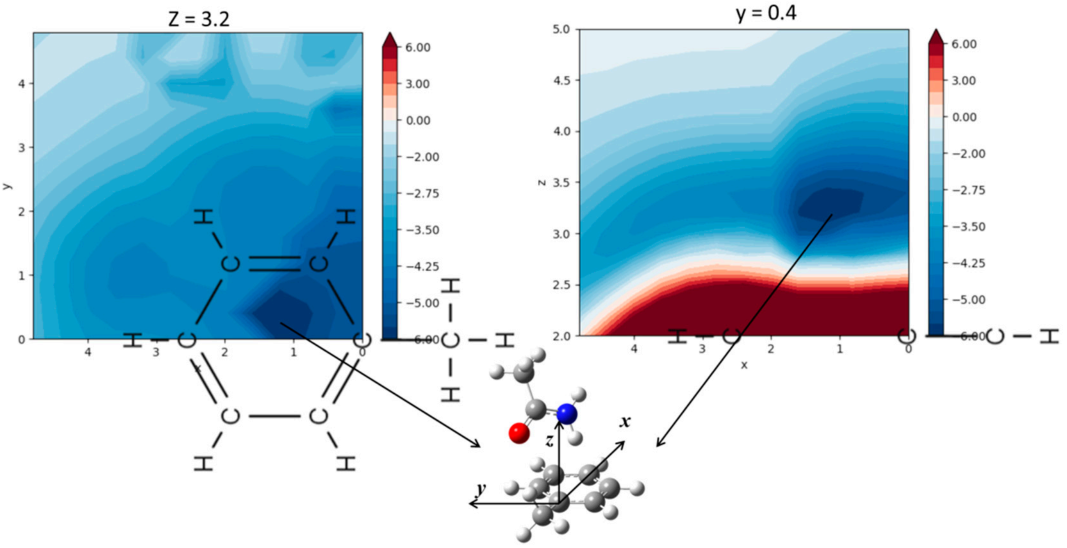

Figure S1. Energy surface of an acetamide-toluene complex with quantum chemical calculation. The intersection of the three-dimensional energy surface on a x,y-plane in the reference frame in left, and a z,x-plane in the reference frame in right.

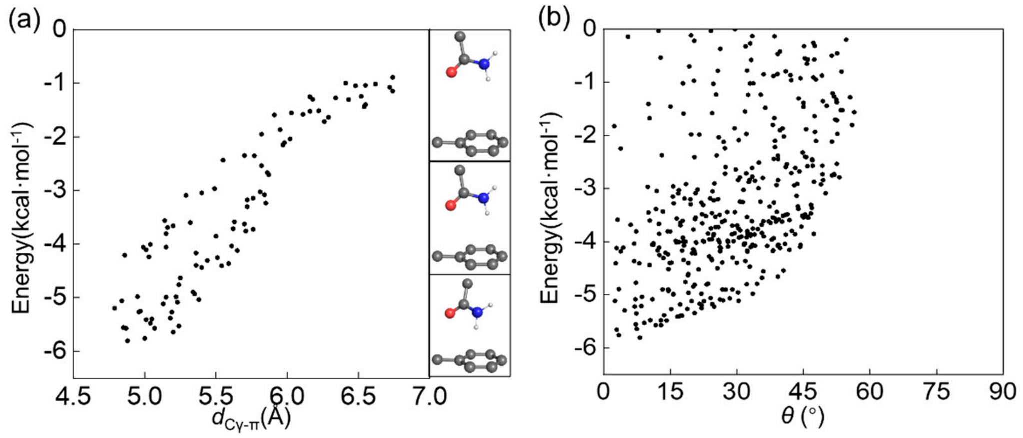

Figure S2. Scatter plot of the data points with (a) 40° < τ < 50° are projected on the ΔE- $d_{C\gamma\text{-}\pi}$ plane and (b) $d_{NM} \leq 4$ Å are projected on the ΔE-θ plane.

Table S1. Terms of interaction energy from S□PT2+3/aug-cc-pVQZ and MP2 methods.

	S□PT (kcal/mol)	MP2 (kcal/mol)
E_{ele}	−4.64	
E_{ee}	7.30	
E_{ind}	−1.67	
E_{disp}	−6.31	
E_{total}	−5.31	−5.72

Table S2. Crystal. Statistics of crystal data collection and structure refinement
One crystal was used for each structure. Values in parentheses are for the highest resolution shell. R_{merge}=ΣhΣi|*Ih,i-Ih*|/ΣhΣi*Ih,i*, where *Ih* is the mean intensity of the *i* observations of symmetry related reflections of *h*. *R*=Σ|F_{obs}-F_{calc}|/ΣF_{obs}, where F_{calc} is the calculated protein structure factor from the atomic model (R_{free} was calculated with 5% of the reflections selected randomly).

	7VEG
**A. Data collection**	
Space Group	$P2_1 2 2_1$
Cell dimensions	
a, b, c (Å)	24.09, 28.01, 73.37
α, β, γ (°)	90, 90, 90
Resolution (Å)	50.00~1.385
R_{merge} (%)	14.1 (41.9)
I / σI	44.5 (4.86)
Completeness (%)	90.62 (23.70)
Redundancy	11.2 (4.3)
**B. Refinement**	
Resolusion (Å)	22.89~1.385
No.reflections	9667（246）
R_{work} / R_{free} (%)[a]	16.7 / 18.0
No. atoms	598
Protein	435
Ligands	48
solvent	115
B-factors	
Protein	15.19
R.m.s.d.[b]	
Bond lengths (Å)	0.011
Bond angles (°)	1.43
Ramachandran plot statistics (%)	
Most favored	100
□dditional allowed	0.0
Generously allowed	0.0
Disallowed	0.0

[a] 10% of reflections were included in the test set.

[b] Root mean square deviation.

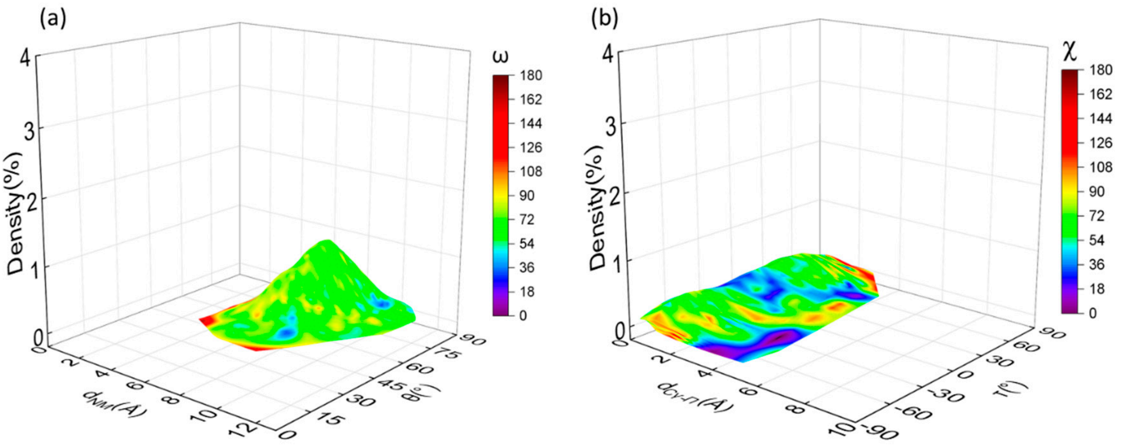

Figure S3. Molecular dynamics simulation of the collagen-like peptide GPQGFO. The geometric parameters of the Q-F pair between the traling and leading chain were extracted from the trajectory and plotted into three-dimensional density maps. (a) Conformation occurring percentage is plotted against d_{NM} and θ. The surface is color-coded with ω. (b) Conformation occurring percentage is plotted against $d_{C\gamma\text{-}\pi}$ and τ. The surface is color-coded with χ. The color scales are located on the right. Insets of (a, b) show the most occurring conformations.

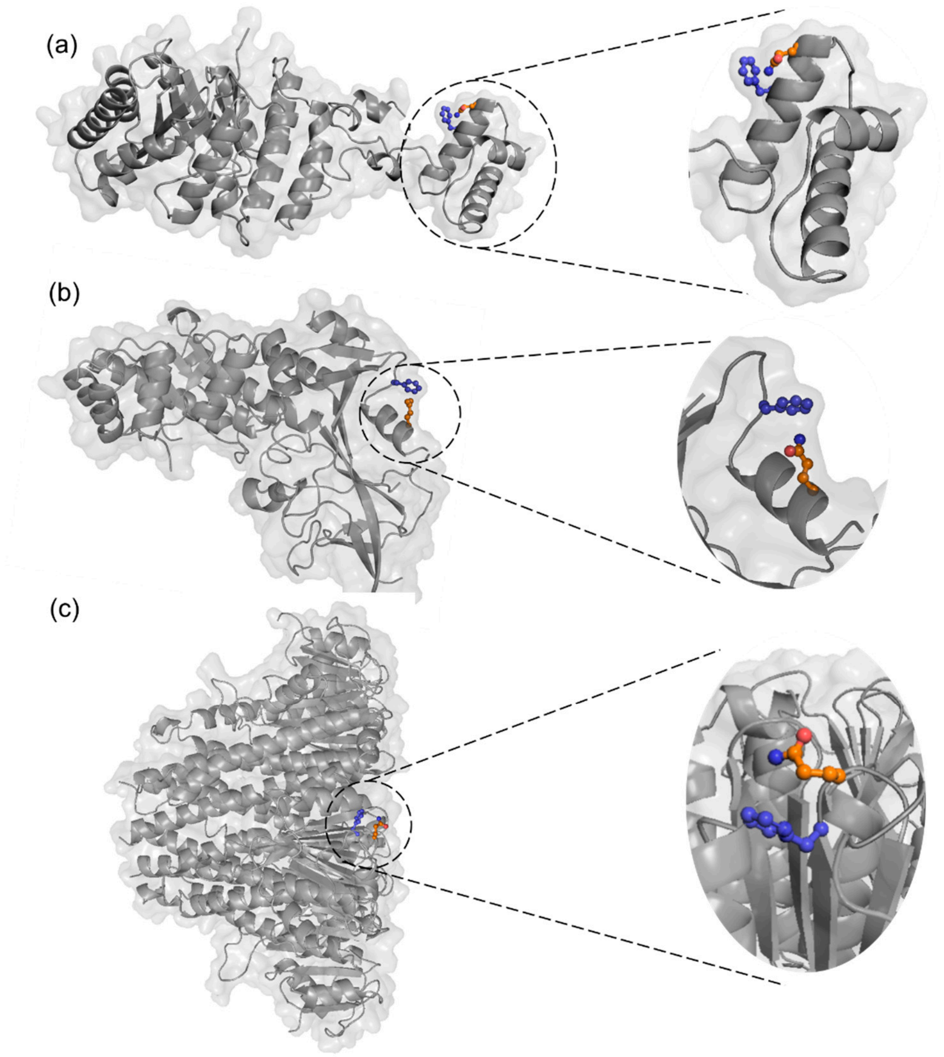

Figure S4. Side chain interactions of Q-F pairs exposed on protein surface in the crystal structure of PDB ID: (a) 4OV4, (b)7ODC, and (c) 3CK6.

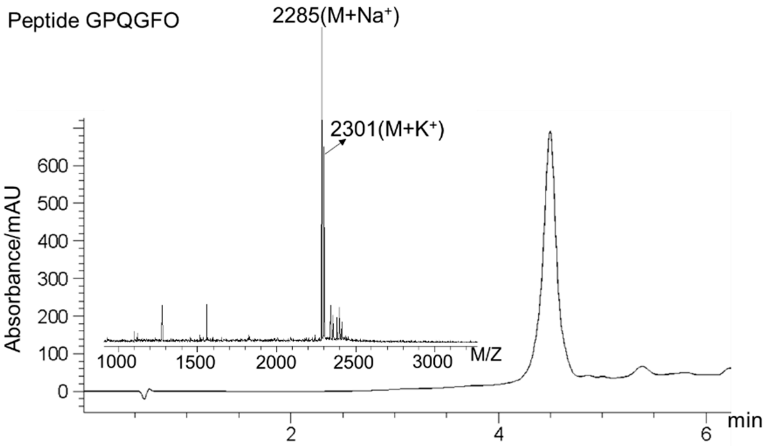

Figure S5. HPLC Chromatograms and Mass Spectrometry analysis of peptides.
Peptide GPQGFO: [M+Na$^+$] expected = 2285.39 / observed = 2285.0

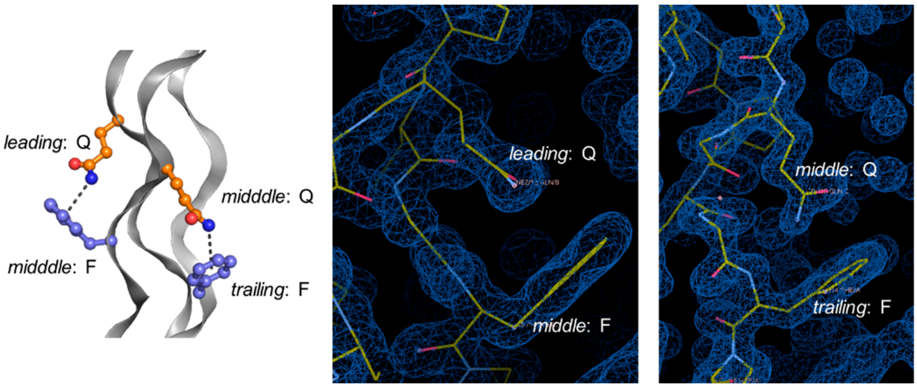

Figure S6. The electron density map of the Q-F pairwise. Figure were generated with COOT.
